# Response: Comment on “Significant decrease in plasma D‐dimer levels and mean platelet volume after a 3‐month treatment with rosuvastatin in patients with venous thromboembolism”

**DOI:** 10.1002/clc.23916

**Published:** 2022-09-08

**Authors:** Toktam Alirezaei, Rana Irilouzadian

**Affiliations:** ^1^ Clinical Research Development Unit of Shohada‐e Tajrish Hospital Shahid Beheshti University of Medical Sciences Tehran Iran

1

We would like to thank Dr. Coban for his interest in our study and for taking the time to express his concerns. The letter has discussed the use of anticoagulants and the method used for measuring mean platelet volume (MPV).

In our study entitled “Significant decrease in plasma D‐dimer levels and mean platelet volume after a 3‐month treatment with rosuvastatin in patients with venous thromboembolism,” we measured MPV using ethylenediaminetetraacetic acid (EDTA). Studies have shown that EDTA and citrate are similar when the time between blood collection and measurement is less than 1 h which was consistent with our study.^1^ Baseline and 3‐month MPV were measured in the same laboratory with the same method. Also, we have considered MPV changes in our data analysis instead of the amount of MPV.^2^, ^3^ Moreover, any previous anticoagulant consumption and antiplatelet use in the last 6 months was an excluding criteria for our study. Also, the same regimen of anticoagulants was used for all of the patients with venous thromboembolism who were included in the study.

Taken all together, we tried to reduce the errors and the factors that may adversely affect our results.
